# *In vitro* and *in silico* analyses of amino acid substitution effects at the conserved N-linked glycosylation site in hepatitis B virus surface protein on antigenicity, immunogenicity, HBV replication and secretion

**DOI:** 10.1371/journal.pone.0316328

**Published:** 2025-01-06

**Authors:** Patcharin Tepjanta, Thammakorn Saethang, Kazuhito Fujiyama, Ryo Misaki, Ingorn Kimkong

**Affiliations:** 1 Department of Microbiology, Faculty of Science, Kasetsart University, Bangkok, Thailand; 2 Department of Computer Science, Faculty of Science, Kasetsart University, Bangkok, Thailand; 3 International Center for Biotechnology (ICBiotech), Osaka University, Osaka, Japan; National Institute of Biologicals (NIB), Ministry of Health & Family Welfare, Government of India, INDIA

## Abstract

The "a" determinant, a highly conformational region within the hepatitis B virus large surface protein (LHBs), is crucial for antibody neutralization and diagnostic assays. Mutations in this area can lead to conformational changes, resulting in vaccination failure, diagnostic evasion, and disease progression. The "a" determinant of LHBs contains a conserved N-linked glycosylation site at N320, but the mechanisms of glycosylation in LHBs remain unclear. This study aimed to investigate the impact of amino acid substitutions at N320 on antigenicity, three-dimensional (3D) structures of LHBs, immunogenic epitopes, and HBV DNA levels. LHBs were mutated by substituting asparagine 320 with proline, cysteine, lysine, and glutamine. The reactivity of the mutants with antibodies was evaluated by western blotting and immunofluorescence staining. Results showed increased binding affinity in N320C, N320Q, and particularly N320P mutants compared to the wild type, likely attributed to conformational changes predicted by the I-TASSER server and further refined by the GalaxyRefine server. Analysis conducted using the IEDB server indicated that the N320P mutation increased the antigenic index, whereas the N320C mutation significantly decreased it. Conversely, the N320K and N320Q mutations exhibited minor effects on antigenicity. Our observations also identified N320P as a potential B-cell epitope and a binding epitope for MHC-I (T-cell epitope). Furthermore, mutating the conserved N-linked glycosylation site at position N320 to proline significantly increased the secretion of HBV DNA in virions. This study enhances our understanding of the impact of a single amino acid mutation at N320 on antibody interaction, LHBs conformation, immunogenicity, and HBV DNA replication. These insights hold promise for advancements in HBsAg detection and the development of vaccines against hepatitis B virus.

## Introduction

Hepatitis B virus (HBV) is the most common infectious agent affecting the liver worldwide. Patients with chronic hepatitis B are particularly at risk of developing cirrhosis and hepatocellular carcinoma (HCC) [1]. According to the World Health Organization, around 296 million individuals were living with chronic HBV infection in 2019, with approximately 1.5 million new infections occurring annually [2].

The HBV virion is enveloped by three virus-encoded surface (envelope) glycoproteins, referred to as the large (LHBs), middle (MHBs), and small (SHBs), which constitute the HBV surface antigen (HBsAg) [3, 4]. These HBV surface proteins are related to each other by sharing a common carboxy-terminal end, corresponding to the S domain (SHBs) [5]. MHBs has an additional pre-S2 domain, whereas LHBs extends MHBs via the pre-S1 polypeptide [6]. In the clinical setting, detection of hepatitis B surface antigen (HBsAg) through serological screening assays is crucial for establishing a diagnosis of HBV infection.

The region between amino acids 99 and 169 of the S domain has been designated as the major hydrophilic region (MHR) and contains several conformational epitopes [7]. The MHR "a" determinant (residues 124–147) is a highly conformational structure composed of two loops spanning residues 124–137 and 139–147, formed by disulfide linkages [8]. Generally, mutations in the “a” determinant induce the generation of HBV immune escape mutants, which can result in the ineffectiveness of HBV vaccination [9]. Several reports have shown that mutations in the region comprising residues 120–147 alter antigenicity and antibody-binding specificity, leading to a failure of HBV detection (diagnostic escape) by conventional diagnostic HBsAg assays [10–14]. Commercially available HBsAg assays must accurately detect samples with known mutations in the “a” determinant region, such as the common vaccine-induced immune-escape variant G145R, to avoid false-negative results [9, 15, 16].

The HBV envelope glycoproteins contain a conserved N-linked glycosylation site at N146 in the S domain (equivalent to N320 of LHBs) and produce two isoforms, glycosylated (gp42) and non-glycosylated (p39) [17]. N-linked glycosylation is essential for HBV virion secretion [18]. This site also plays a protective role against neutralizing antibodies, while un-glycosylation appears essential for infectivity [19]. Variants with amino acid substitutions at this N-linked glycosylation site have been identified in patients with chronic HBV infection [3, 19–21]. Removal of this site prevents the secretion of virions but does not affect the synthesis and stability of HBsAg [3, 22]. Due to failures in both diagnosis and vaccination, individuals who are infected may become chronic silent carriers because the virus persists in hepatocytes and peripheral blood. However, this outcome can be averted by the development of new diagnostic assays and vaccine strategies [23].

Immunoinformatics is a branch of bioinformatics dedicated to studying the relationship between immune responses and predicted epitopes [24]. The identification and prediction of antigenic regions for specific epitopes recognized by B-cells and T-cells through MHC molecules are among the most important applications of immunoinformatics [25]. Immunoinformatics serves as a valuable tool for identifying new antigenic epitopes, facilitating the design of vaccines against diverse infectious diseases caused by various pathogens, including bacteria, viruses, fungi, and parasites [26]. This method offers cost and time savings compared to laboratory tests [25].

Specific modifications of glycoproteins have been used as biomarkers and therapeutic targets for various diseases, including cancers and infectious diseases [27]. Therefore, investigating the evolution of a single amino acid substitution at the conserved N-linked glycosylation position, N320, of LHBs (equivalent to N146 of SHBs), would be of interest. In this study, we examined whether modification of a conserved N-linked glycosylation site (N320) in LHBs by amino acid substitution affects their antigenicity properties, the 3D conformation of LHBs, particularly concerning "a" determinant-containing mutants, immunogenic epitopes, and HBV DNA levels. This research contributes to understanding the role of N-linked glycosylation in the biology of HBV infection and has implications for the development of novel HBV diagnostic assays and vaccines.

## Materials and methods

### Plasmid construction

The HBs ORF utilized in this study was synthesized based on the HBV genome sequence (GenBank accession no. X02763.1, subtype *adw2*) and fused with an amino-terminal polyhistidine tag (His6-tag). HindIII and NheI restriction sites were incorporated into the primer sequences for cloning purposes. The PCR product underwent purification and was subsequently cloned into a pCDNA-3.1(+) vector, resulting in the creation of the plasmid pLHBs-WT.

### Site-directed mutagenesis

Primers containing the N320P, N230C, N320K, and N320Q mutations were utilized to introduce these mutations into the pLHBs-WT construct. The mutants were generated using the QuikChange II site-directed mutagenesis kit following the manufacturer’s instructions. In brief, after amplification of the mutated plasmid, the DNA was digested with DpnI and transformed into *E*. *coli* XL1-Blue supercompetent cells. Transformants were selected on Luria-Bertani (LB) plates containing ampicillin (100 μg/ml). Mutated plasmid DNA was isolated from several transformants and identified through sequence analysis. Four new plasmids (pLHBs-N320P, pLHBs-N320C, pLHBs-N320K, and pLHBs-N320Q) were generated.

### Cell culture and transient transfection

The human embryonic kidney cell line HEK-293T, provided by Associate Professor Nakarin Kitkumthorn from the Faculty of Dentistry, Mahidol University, was cultured in Dulbecco’s Modified Eagle’s medium (DMEM; Gibco, USA) supplemented with 10% heat-inactivated fetal bovine serum (FBS; Gibco, USA) and 1% penicillin-streptomycin (10000 U/ml, Gibco, USA). The HBV-replicating hepatoma cell line HepG2.2.15, provided by Professor Nattiya Hirankarn from the Faculty of Medicine, Chulalongkorn University, was cultured in RPMI-1640 medium (Gibco, USA) supplemented with 10% FBS (Gibco, USA) and 380 μg/mL geneticin G418 (Toku-E, USA). All cell lines were maintained at 37°C in a humidified chamber supplemented with 5% CO_2_. For experiments, cells were seeded at a density of 6.0 × 10^5^ cells per well in six-well plates and transfected 18–24 hours later, when the cell density reached approximately 80%. Transfection of constructed plasmids was carried out using Lipofectamine™ 3000 Transfection Reagent (Invitrogen, USA), following the manufacturer’s instructions. Transfection efficiency was assessed based on GFP expression in the cells.

### Western blot analysis

Transfected HEK-293T cells were scraped and lysed using the CytoBuster^TM^ protein extraction reagent (Merck, Germany) in the presence of protease and phosphatase inhibitor cocktail tablets (Roche, Switzerland). Protein concentrations were determined using a Pierce BCA Protein Assay Kit (Thermo Fisher Scientific, USA), according to the manufacturer’s instructions. For western blot analysis, 20 μg of soluble protein lysates were separated by 12% SDS-PAGE and transferred to polyvinylidene fluoride (PVDF) membranes (Merck, Germany). After blocking with 5% skimmed milk, membranes were probed with Anti-Hep B preS1 (1:1000; sc-57762; Santa Cruz Biotechnology, USA)) at 4°C overnight followed by horseradish peroxidase (HRP)-conjugated mouse IgGκ-binding protein (1:5000; sc-516102; Santa Cruz Biotechnology, USA) at room temperature for 1 hour. Chemiluminescent signals of the bound HRPs on the membranes were detected using a SuperSignal West Femto substrate (Thermo Scientific, USA) in a UVITEC Alliance Q9 Advanced imager (Uvitec Ltd., UK). The amount of protein was normalized to that of GAPDH (sc-47724; Santa Cruz Biotechnology, USA). The optical density of the bands from the protein blots was quantified using the ImageJ software (National Institutes of Health, USA).

### Immunofluorescence staining

HEK-293T cells were cultured in eight-well chamber slides (Thermo Scientific, USA) and transfected with pLHBs-WT or mutant plasmids. Forty-eight hours post-transfection, the cells were washed with phosphate-buffered saline (PBS) and fixed with 4% paraformaldehyde for 30 minutes at room temperature. The fixed cells were washed thrice with PBS, permeabilized with 0.1% Triton X-100 in PBS for 30 minutes and blocked with 1% bovine serum albumin (BSA) for 30 minutes. Subsequently, the cells were incubated with diluted anti-Hep B preS1 antibody (1:200; sc-57762; Santa Cruz Biotechnology, USA) containing 0.1% BSA. After overnight incubation at 4°C, the cells were washed thrice with PBS. Following that, the cells were incubated with mouse-IgGκ BP-CFL 488 binding protein (1:50; sc-516176; Santa Cruz Biotechnology, USA) at room temperature for at least 1 hour. The cell nuclei were stained with DAPI, and the slides were mounted with ProLong Gold Antifade Mountant (Thermo Fisher Scientific, UK). Images were captured using an Olympus FV 3000 confocal microscope and analyzed with Olympus FV31S-SW (Ver.2.6) software.

### Extraction of replicative HBV DNA from core particles and virion DNA from culture supernatant

The established protocol was used to extract replicative DNA associated with core particles and HBV DNA from secreted virions [28]. Briefly, HepG2.2.15 cells were lysed with lysis buffer (50 mM Tris‐HCl, pH 7.4, 150 mM NaCl, 5 mM MgCl_2_, and 1% NP‐40) for 10 minutes. The cytoplasmic fraction was separated from the nuclear fraction through centrifugation. Supernatants were then transferred to a new tube, mixed with 10 mM MgCl_2_ and 500 μg/ml DNase I (Sigma-Aldrich, USA), and incubated for 1 hour at 37°C. The reaction was stopped with 25 mM EDTA. Subsequently, sodium dodecyl sulfate and proteinase K (Vivantis, Malaysia) were added separately and incubated for 1.5 hours at 56°C. Viral DNA was then purified through phenol-chloroform extraction and isopropanol precipitation. HBV progeny DNA was extracted from virions in the cell culture supernatant using a QiAamp DNA blood mini kit (Qiagen, Germany). The detection of HBV DNA was performed using quantitative real-time PCR (qPCR), and the quantification of HBV DNA was determined by comparison to a standard curve.

### Evaluation of the mutations on the conformation of LHBs

The amino acid sequences of the wild-type and mutant types of LHBs were subjected to the I-TASSER server for "ab initio" prediction of their structures. The I-TASSER software, available at https://zhanggroup.org/I-TASSER/, utilizes multiple-threading alignments and iterative template fragment assembly simulations methods for modeling protein structure [29]. The resulting models were refined by the GalaxyRefine server (http://galaxy.seoklab.org/cgi-bin/submit.cgi?type=REFINE2) [30]. The best refined model was evaluated based on the results of the Ramachandran plot obtained from the VADAR server (http://vadar.wishartlab.com/) [31]. he obtained protein 3D structures were visualized using the UCSF Chimera program [32].

### Evaluation of protein antigenicity

It has been reported that amino acid substitutions both outside and within “a” determinant of HBsAg are associated with a reduced binding affinity of the antigen to monoclonal antibodies developed against the “a” determinant [33]. To further understand the immunogenic potential of LHBs mutants, the mutant sequences were submitted to the antibody epitope prediction server IEDB-AR [34].

### Identification of the B-cell epitopes

The goal of B-cell epitope prediction is to determine the antigen recognized by B lymphocytes and initiate humoral immunity. The ABCpred server (https://webs.iiitd.edu.in/raghava/abcpred/index.html) [35] and the IEDB B-cell epitope prediction tool (http://tools.iedb.org/bcell/) were used to identify the linear B-cell epitopes within the LHBs-N320P sequence. The IEDB B-epitope prediction server includes BepiPred linear epitope prediction [36–38], Chou–Fasman beta-turn prediction [39], Emini surface accessibility prediction [40], Karplus–Schulz flexibility prediction [41], Kolaskar–Tongaonkar antigenicity [42], and Parker hydrophilicity prediction [43].

### T-cell epitope prediction

MHC allele-peptide binding serves as the primary basis for predicting potential T-cell epitopes. Therefore, the LHBs-N320P sequence was screened for MHC-restricted T-cell epitopes using the most precise and reliable T-cell epitope prediction tools (Table 1). Epitopes containing a mutation at N320P with high scores were selected from the outputs of all the servers. Notably, the HLA-A0201 allele, widely investigated for peptide binding, was employed to identify MHC-I binding epitopes.

**Table 1:** List of deep learning-based MHC-peptide binding prediction tools.

Servers	Link	Description
MHC-I processing predictions	http://tools.immuneepitope.org/processing/	identify MHC-I ligands
SYFPEITHI	http://www.syfpeithi.de/bin/MHCServer.dll/EpitopePrediction.htm	Based on motif binding to MHC class I and II
MHC-NP	http://tools.immuneepitope.org/mhcnp/	predict peptides naturally processed by the MHC pathway
RANKPEP	http://imed.med.ucm.es/Tools/rankpep.html	Predicts peptide binders to MHC I and MHC II molecules using position specific scoring matrices (PSSMs)

### Immunogenicity prediction

Immunogenicity prediction tools were used to determine the ability of the epitope/MHC complex to elicit an immune response. The IEDB class I immunogenicity server was designed to predict the immunogenicity of the peptide-MHC complex (http://tools.immuneepitope.org/immunogenicity/) [44]. Epitopes predicted using the method described above were then assessed for immunogenicity, using default parameters. Epitopes with positive values were selected for conservation analysis.

### Statistical analysis

Each experiment was conducted a minimum of three times, and the data are presented as the mean ± standard error of the mean (SEM). Statistical analysis was carried out using the Statistical Product and Service Solutions (SPSS) software (version 25.0; IBM Corporation, USA). Significant differences were determined using Student’s t-test or one-way analysis of variance (ANOVA), followed by Tukey’s multiple comparison test. Statistical significance was set at a P level of <0.05.

## Results

### Mutations at the N-linked glycosylation site (N320) might alter antigenicity of LHBs

**Fig 1:**
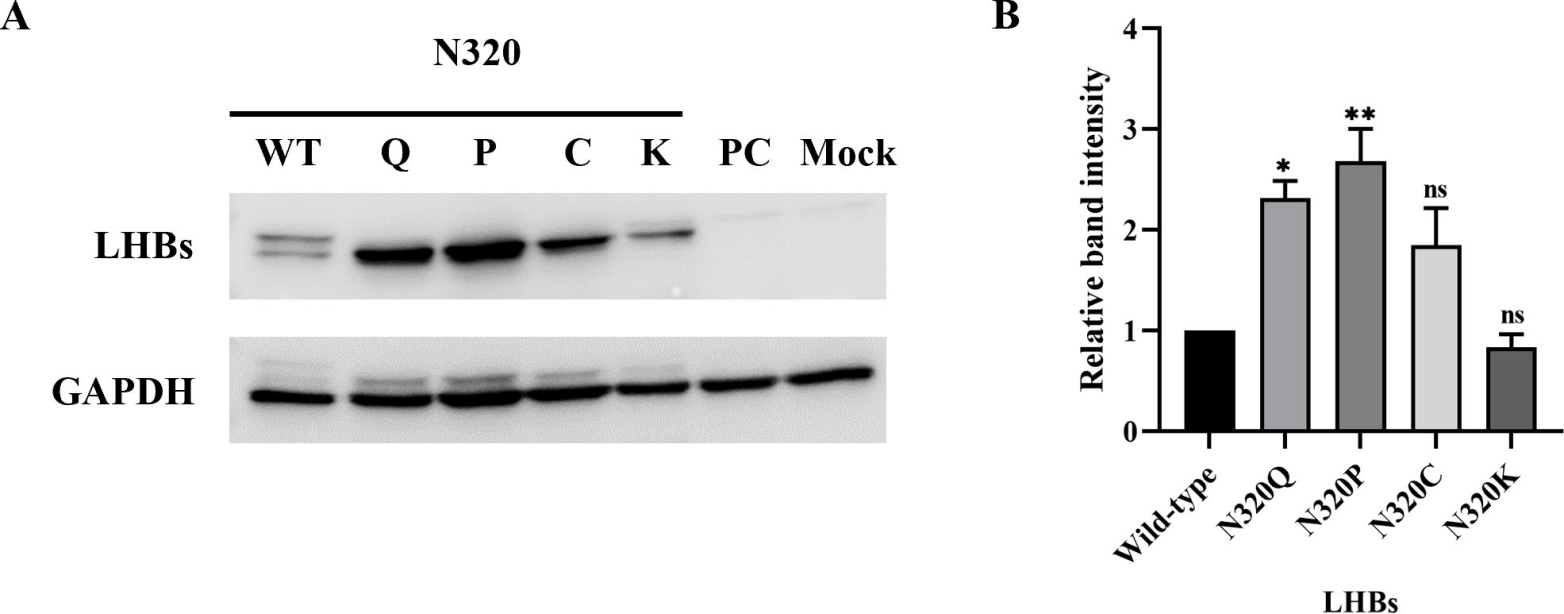
Effect of LHB-specific substitution site at N320 on protein expression. (A) The antigenic reactivity of LHBs was determined using western blotting. (B) The relative band intensities of the LHBs obtained from western blotting were quantified. *p< .05, **p< .01, ***p< .001, ns; not significant.

**Fig 2:**
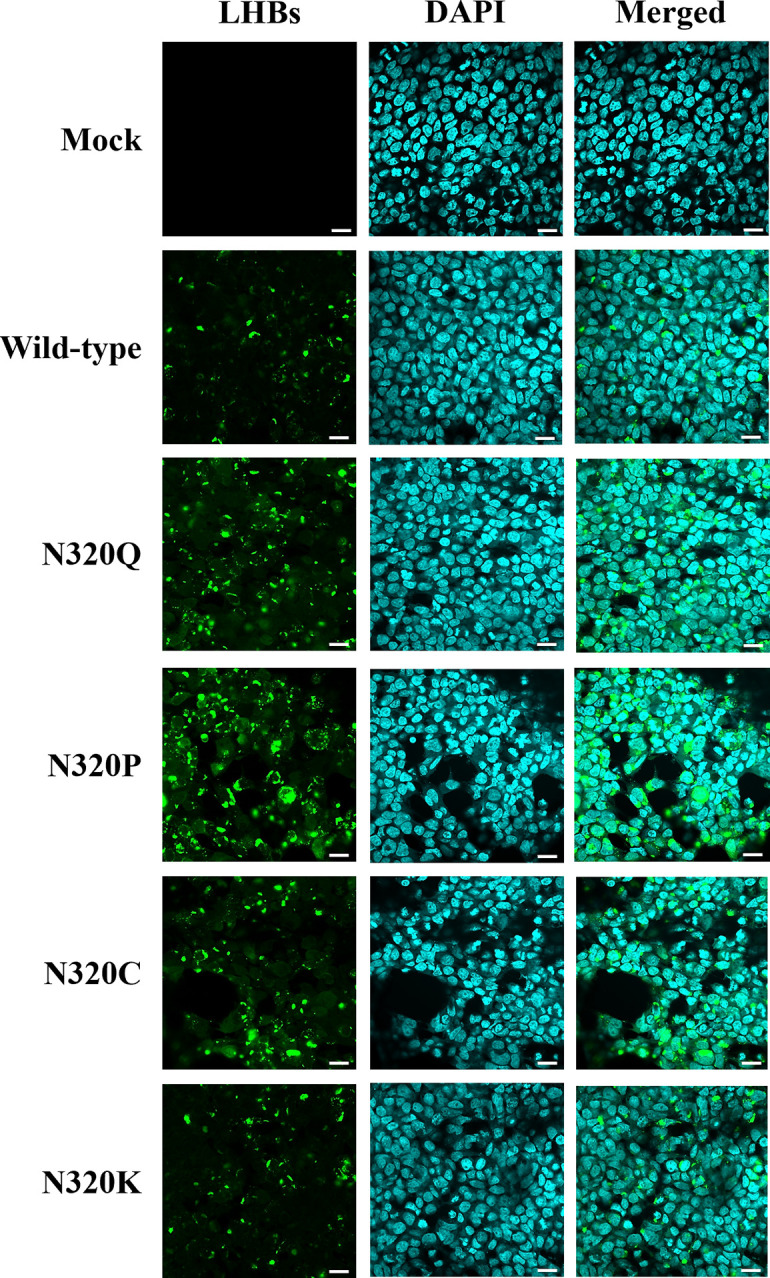
Immunofluorescence staining. The cells on the culture slides were fixed 48 hours after transfection and stained with an anti-Hep B preS1 antibody, followed by incubation with mouse IgGκ binding protein (m-IgGκ BP) conjugated to CruzFluor™ 488 (CFL 488). The LHBs protein and preS1 antibody reactivity is depicted in green, while the cell nuclei stained with DAPI are shown in blue. Cells transfected with the pcDNA3.1(+) plasmid were used as a negative control (mock). The scale bar represents 20 μm.

In our investigation, we examined whether mutations, specifically amino acid substitutions at N320, altered the binding specificity of the anti-preS1 antibody. Initially, we replaced the asparagine residues at position 320, an N-linked glycosylation site situated within the "a" determinant, with proline, cysteine, lysine, and glutamine amino acids. We then analyzed the expression of viral proteins *in vitro* through transient transfection into HEK-293T cells. While both glycosylated (gp) and non-glycosylated (p) forms of wild-type LHBs (gp42, p39) were detected, the LHBs mutants were exclusively observed in the non-glycosylated (p39) form. Remarkably, the LHBs-N320P mutant exhibited significantly increased binding capacity with the preS1 antibody, followed by the LHBs-N320Q and LHBs-N320C mutants, respectively, compared to the wild-type. Conversely, the LHBs-N320K mutant displayed reduced reactivity with the preS1 antibody compared to the wild-type (Fig 1). To validate these findings, we analyzed the expression and distribution of these viral mutant proteins in HEK-293T cells using immunofluorescence staining. The results indicated that the LHBs-N320P mutant was well-expressed in HEK-293T cells, comparable to all mutant and wild-type transfected cells, followed by the LHBs-N320Q and LHBs-N320C mutants. However, the LHBs-N320K mutant demonstrated lower expression levels in HEK-293T cells compared to all mutants, but it was not significantly different from the wild-type (Fig 2). These observations suggest that amino acid mutations in the "a" determinant region at the N-linked glycosylation site (N320) might impact the antigenicity of LHBs.

### Mutation at N320 induces local conformation change in “a” determinant region of LHBs

**Fig 3:**
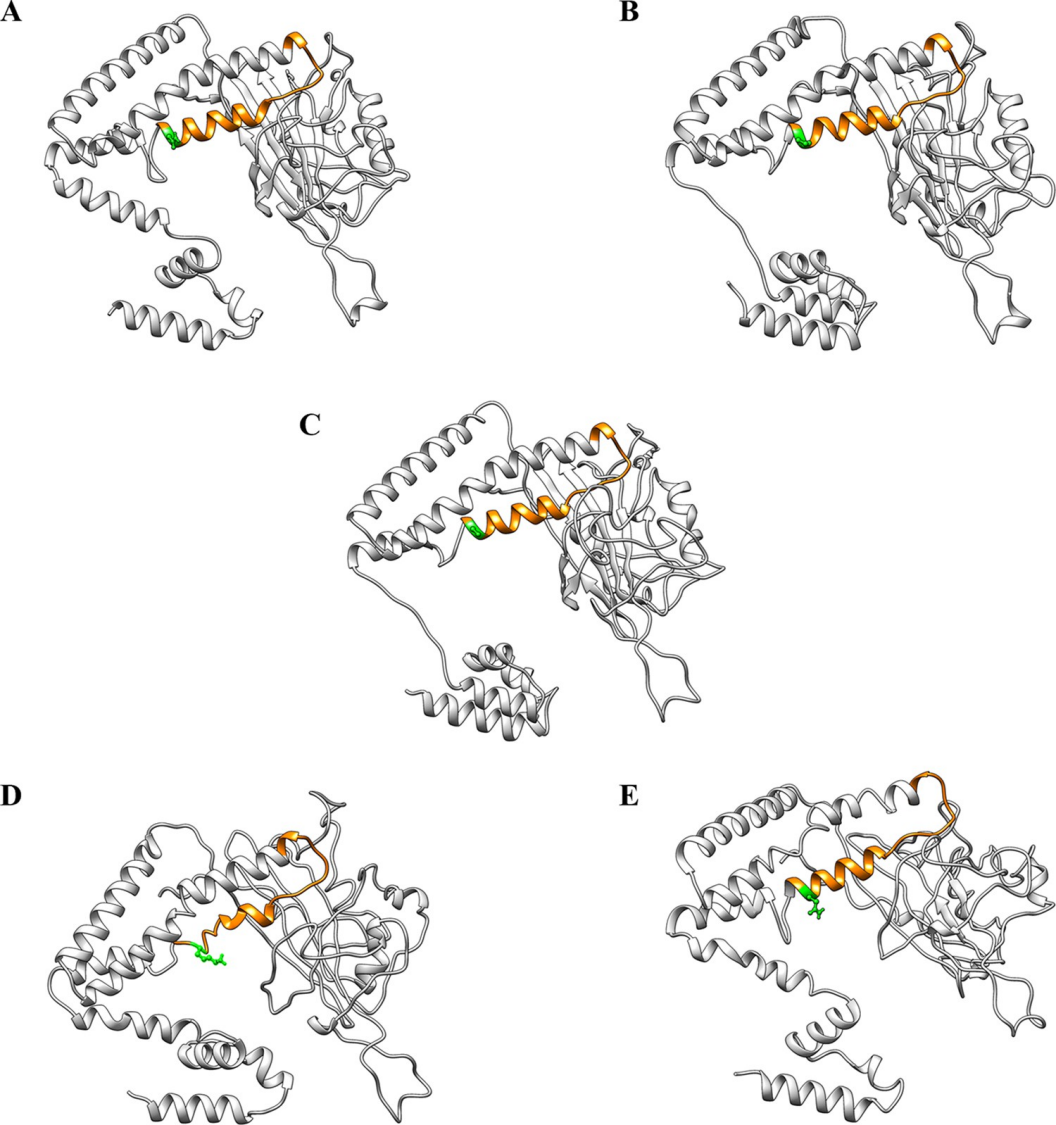
Effect of the LHBs-specific substitution site at N320 on the 3D structure of the protein. Predicted protein 3D structure of (A) wild-type LHBs, (B) LHBs-N320P, (C) LHBs-N320C, (D) LHBs-N320K, and (E) LHBs-N320Q were modeled using the I-TASSER server and refined using the GalaxyRefine server. The “a” determinant region is shown in orange and substituted residues are shown in green with balls and sticks.

**Fig 4:**
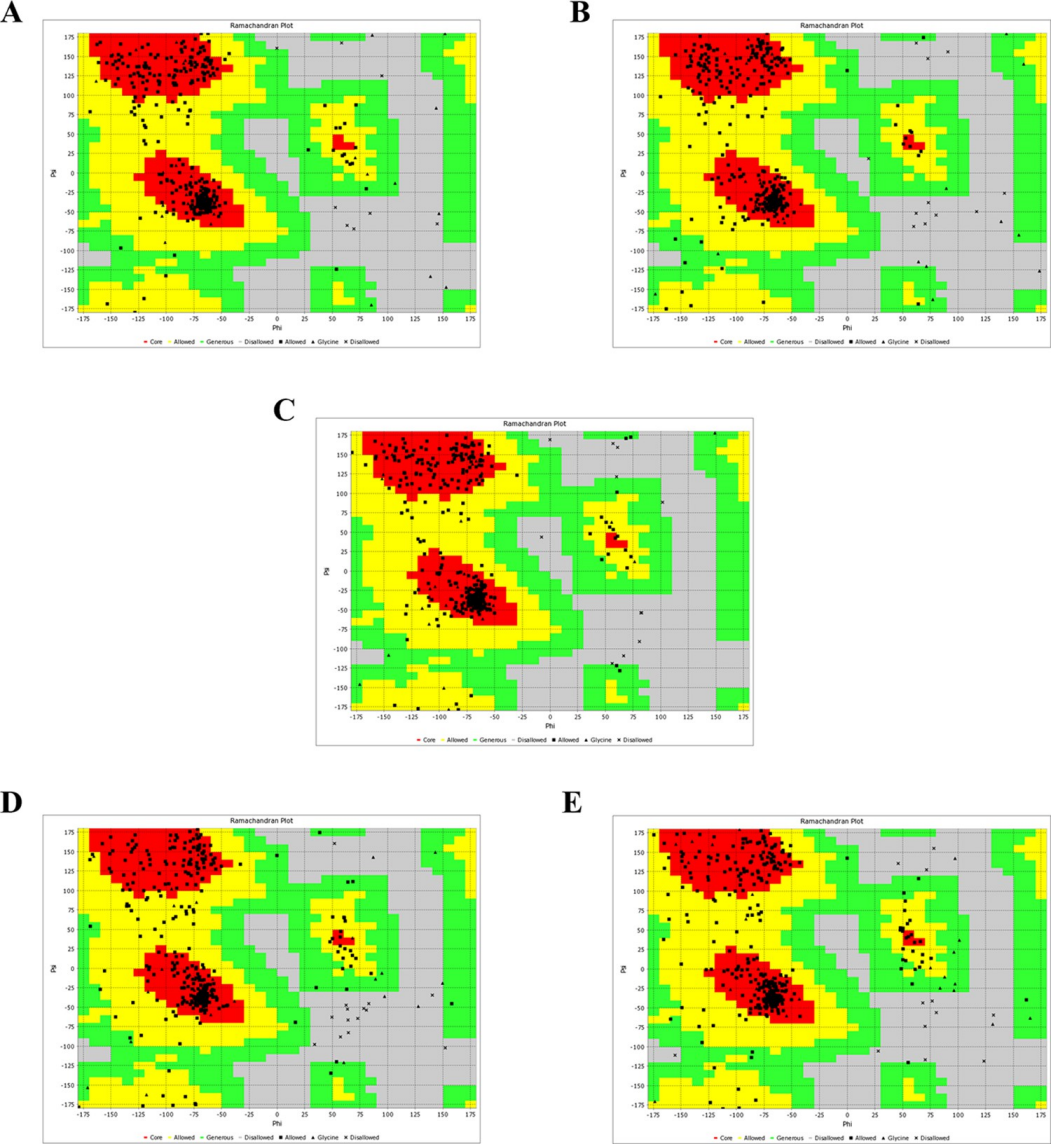
Evaluation of the LHBs 3D model quality based on the Ramachandran plot. Ramachandran plots of (A) wild-type LHBs, (B) LHBs-N320P, (C) LHBs-N320C, (D) LHBs-N320K, and (E) LHBs-N320Q derived from the VADAR program. Different regions, including the core, allowed, generous, and outside regions, are shown in red, yellow, green, and gray, respectively.

To demonstrate the impact of amino acid mutations within the "a" determinant region at the N-linked glycosylation site (N320) on the antigenicity of LHBs, potentially through alterations in their conformation, we modeled the 3D structures of the wild-type and mutant LHBs using the I-TASSER server, followed by refinement with the GalaxyRefine server. Our results revealed that the conformational changes induced by all mutations differed from those in the wild-type protein, particularly manifesting as local alterations in the "a" determinant region. Notably, the N320K mutation exerted a more substantial effect on the conformation of the "a" determinant. In addition to inducing local changes in the "a" determinant, the N320K mutation led to a transformation of the alpha-helix into a non-repetitive secondary structure in the "a" determinant region compared to wild-type LHBs (Fig 3). Furthermore, we employed a Ramachandran plot, a tool for assessing the quality of protein structures, to validate the refined predicted models obtained from the GalaxyRefine server. Analysis of the Ramachandran plot revealed that most residues in all of the obtained 3D structures were located in the most favored (phi psi core) region, indicated in red, signifying an acceptable 3D model (Fig 4). Detailed Ramachandran plot analyses of the LHBs protein-predicted models are provided in Table 2. The distribution of residues in the core, allowed, generous, and outside regions of the wild-type LHBs model was 86%, 11%, 1%, and 2%, respectively. Additionally, the distribution of residues in the core, allowed, generous, and outside regions of the LHBs-N320P model was 85%, 12%, 1%, and 2%, respectively; the LHBs-N320C model was 85%, 11%, 2%, and 2%, respectively; the LHBs-N320K model was 77%, 11%, 3%, and 3%, respectively; and the LHBs-N320Q model was 79%, 15%, 3%, and 3%, respectively. Taken together, these results demonstrate that the 3D structures of the wild-type and mutant LHBs obtained from the I-TASSER server and refined by the GalaxyRefine server are acceptable, as confirmed by a Ramachandran plot.

**Table 2:** Ramachandran plot details of the LHBs protein-predicted models.

LHBs	Core region (%)	Allowed region (%)	Generous region (%)	Outside region (%)
Wild-type	86	11	1	2
N320P	85	12	1	2
N320C	85	11	2	2
N320K	77	17	3	3
N320Q	79	15	3	3

### Mutations at N320 cause changes in the antigenicity prediction of LHBs

**Fig 5:**
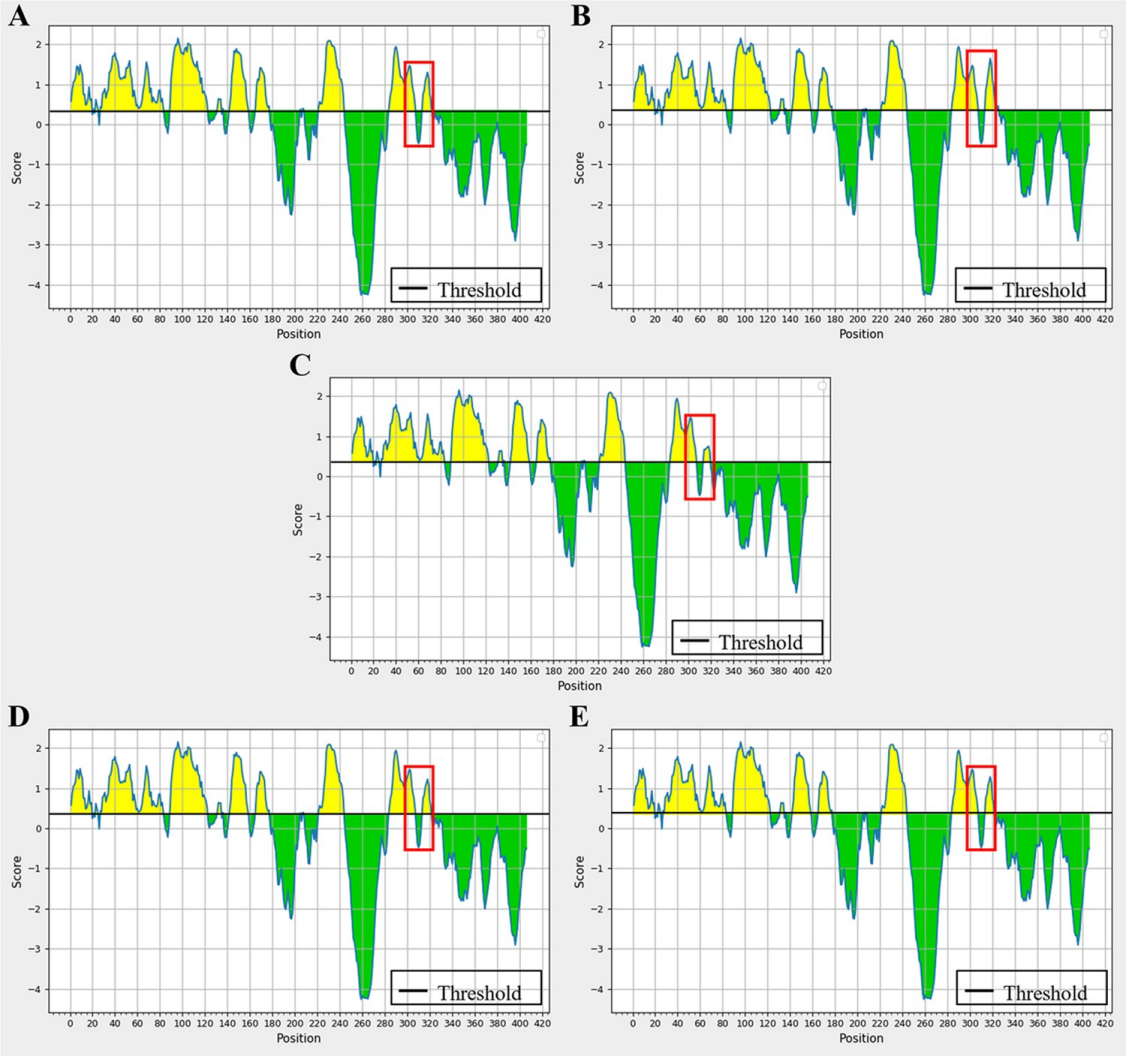
Antigenicity analysis of the LHBs variants. (A) wild-type LHBs, (B) LHBs-N320P, (C) LHBs-N320C, (D) LHBs-N320K, and (E) LHBs-N320Q. Antigenic and non-antigenic segments are shown in yellow and green, respectively. The “a” determinant is indicated by the open boxes.

The prediction of the antigenicity of mutant sequences with a single amino acid substitution at N320 within the "a" determinant of LHBs revealed changes in antigenicity at and around the site of the amino acid substitution compared to the wild-type sequence (Table 3). Specifically, for the N320P substitution, several amino acids at and around the site exhibited remarkably increased antigenicity (+0.087 to +0.342) compared to the wild-type sequence. Conversely, notably reduced antigenicity was observed with the N320C substitution (-0.087 to -0.533) compared to the wild-type sequence. Meanwhile, the amino acid substitutions at N320K and N320Q showed only slight changes in antigenicity (-0.008 to -0.075) compared to the wild-type sequence. Moreover, the results obtained from the IEDB server indicated that the LHBs-N320P mutant displayed a moderate increase in the antigenic properties of LHBs compared to the wild-type sequence (Fig 5B). However, the LHBs-N320C mutant exhibited a moderate reduction in LHBs antigenic properties compared to the wild-type sequence (Fig 5C). These findings collectively suggest that amino acid substitution at the N-linked glycosylation site N320 within the "a" determinant region can influence the antigenicity of LHBs.

**Table 3:** The effects of the LHBs-specific substitution site at N320 on antigenicity.

Wild-type LHBs		LHBs-N320P		LHBs-N320C		LHBs-N320K		LHBs-N320Q	
Positions	Score	Positions	Score	Positions	Score	Positions	Score	Positions	Score
S310	-0.48	S310	-0.48	S310	-0.48	S310	-0.48	S310	-0.48
C311	-0.406	C311	-0.406	C311	-0.406	C311	-0.406	C311	-0.406
C312	-0.134	C312	-0.134	C312	-0.134	C312	-0.134	C312	-0.134
C313	0.329	C313	0.329	C313	0.329	C313	0.329	C313	0.329
T314	0.758	T314	0.845	T314	0.671	T314	0.75	T314	0.758
K315	0.843	K315	1.011	K315	0.666	K315	0.825	K315	0.841
P316	1.084	P316	1.24	P316	0.702	P316	1.027	P316	1.061
T317	1.194	T317	1.441	T317	0.728	T317	1.128	T317	1.175
D318	1.303	D318	1.645	D318	0.75	D318	1.227	D318	1.286
G319	1.16	G319	1.502	G319	0.608	G319	1.085	G319	1.143
N320	**0.753**	P320	**1.095**	C320	**0.201**	K320	**0.678**	Q320	**0.736**
C321	0.609	C321	0.952	C321	0.057	C321	0.534	C321	0.592
T322	0.262	T322	0.604	T322	-0.291	T322	0.187	T322	0.245
C323	0.087	C323	0.343	C323	-0.378	C323	0.02	C323	0.071
I324	0.168	I324	0.342	I324	-0.207	I324	0.111	I324	0.154
P325	0.281	P325	0.467	P325	0.11	P325	0.262	P325	0.286
I326	0.126	I326	0.221	I326	0.04	I326	0.117	I326	0.128
P327	0.188	P327	0.188	P327	0.188	P327	0.188	P327	0.188
S328	0.026	S328	0.026	S328	0.026	S328	0.026	S328	0.026
S329	0.234	S329	0.234	S329	0.234	S329	0.234	S329	0.234
W330	0.167	W330	0.167	W330	0.167	W330	0.167	W330	0.167

### Peptide sequences containing the N320P mutation could potentially serve as B-cell epitope

In the current study, the mutation of asparagine to proline at the N-linked glycosylation site 320 resulted in an enhanced antigenic index in the vicinity of the mutation site compared to the wild-type sequence. Subsequently, we explored its potential as a B-cell epitope, particularly at the position N320P. The linear B-cell epitopes were identified using the ABCpred server and IEDB B-cell epitope prediction tool. The predicted peptides containing the N320P mutation, which received the highest score according to the ABCpred tool, were selected and further analyzed using the IEDB prediction tool to enhance the accuracy of epitope prediction. The findings from ABCpred revealed that peptide sequences spanning residues 309–324 and 312–329 exhibited high scores of 0.87 and 0.78, respectively. Additionally, each IEDB prediction tool provided a chart showing epitopic regions categorized as positive (with scores higher than the threshold) and negative (with scores lower than the threshold) (Table 4). These results collectively indicate that the N-linked glycosylation mutation at N320P may serve as a pivotal immunogenic B cell-binding epitope.

**Table 4:** The list of B-cell epitopes contains the N320P mutation.

Linear epitopes	(1)	(2)	(3)	(4)	(5)	(6)	(7)	(8)
PSCCCTKPTDGPCTCI	+	+	+	+	+	+	-	0.87
CCTKPTDGPCTCIPIPSS	+	+	+	+	+	+	-	0.78

(1), Antigenicity; (2), Beta turn; (3), Flexibility; (4), Hydrophilicity; (5), Surface accessibility; (6), BepiPred 1.0; (7), BepiPred 2.0; (8), ABCpred score.

### Peptide sequences containing the N320P mutation could potentially serve as T-cell epitope

Based on the HLA-A0201 allele, potential MHC-I binding epitopes were predicted using the IEDB recommendations, SYFPEITHI, MHC-NP, and RANKPEP servers for the LHBs-N320P sequence. However, methods for predicting MHC-I peptide binding are specific to a fixed peptide length. Therefore, most MHC-I peptide ligands consist of nine residues, making models for the prediction of 9-mer binders preferable. In total, 9 potential epitopes containing a mutation at N320P were selected. Results are shown in Table 5. Taken together, these results indicate that the N-linked glycosylation mutation at N320P may also serve as a pivotal immunogenic T-cell binding epitope.

**Table 5:** The list of T-cell epitopes contains the N320P mutation.

Number	Sequence	IEDB Recommend (Total Score)	SYFPEITHI (Score)	MHC-NP (Prob Score)	RANKPEP (Score)
1	CCTKPTDG**P**	-4.05	3	0.0001	-27
2	CTKPTDG**P**C	-3.96	5	0.0017	-22
3	TKPTDG**P**CT	-3.84	5	0.0004	-36
4	KPTDG**P**CTC	-3.53	5	0.0031	-12
5	PTDG**P**CTCI	-3.28	12	0.0009	9
6	TDG**P**CTCIP	-4.31	4	0.0001	-41
7	DG**P**CTCIPI	-3.83	7	0.0002	-20
8	G**P**CTCIPIP	-4.09	6	0	-31
9	**P**CTCIPIPS	-5.12	-4	0.0001	-34

### Immunogenicity prediction

In addition to epitope prediction, further analysis of the binding affinity between the peptide/MHC complex and TCR is crucial. A high immunogenicity score indicates a strong ability to stimulate naive T-cells and induce cellular immunity. The T-cell epitopes selected using the above methods underwent immunogenicity prediction using the IEDB tool. The results revealed that six peptides containing the N320P mutation yielded positive scores (Table 6). The immunogenicity scores of the epitopes ranged from 0.18335 to 0.01614. Additionally, comparison of the final T-cell epitopes with linear B-cell epitopes showed overlaps, suggesting the presence of some T-cell epitopic peptides within B-cell epitopic peptides.

**Table 6:** Immunogenicity of the T-cell epitope contains the N320P mutation.

Number	Epitopes	Immunogenicity Score
1	**P**CTCIPIPS	0.18335
2	G**P**CTCIPIP	0.16274
3	TKPTDG**P**CT	0.0481
4	DG**P**CTCIPI	0.03504
5	KPTDG**P**CTC	0.03466
6	TDG**P**CTCIP	0.01614

### Mutation of N320-linked glycans alters HBV DNA replication and secretion

**Fig 6:**
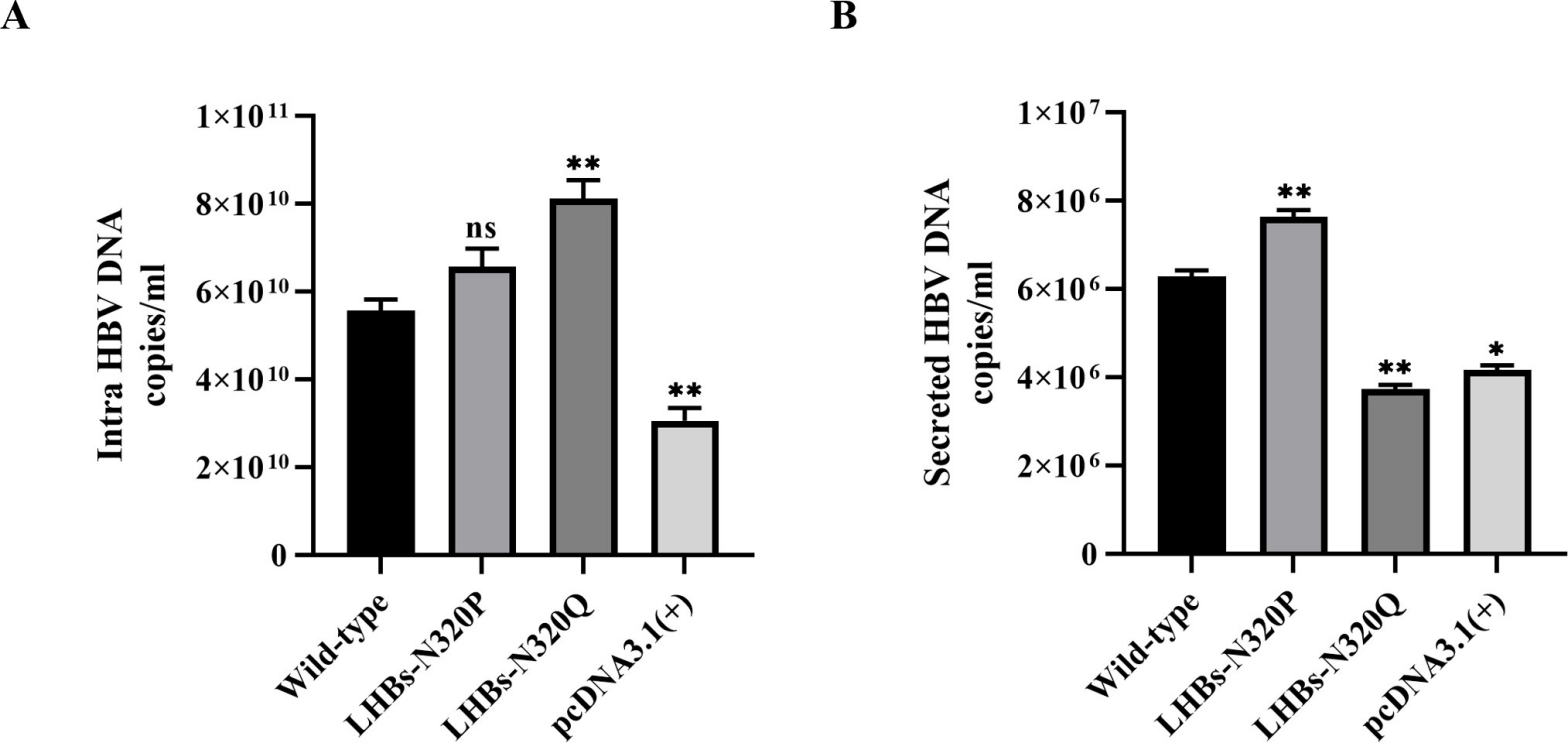
Impact of N-linked glycosylation mutation at position N320 on HBV DNA replication and virion secretion. (A) HBV replicative intermediates were extracted from core particles of transfected HepG2.2.15 cells. (B) HBV DNA was extracted from the secreted virions in the culture supernatants. Qualitative HBV DNA was examined using real-time PCR. The data are plotted as mean and SEM. *p < .05; **p < .01; ***p < .001; ns, not significant.

Mutations or variants within the S/P region, as well as the immunodominant "a" determinant region, have been demonstrated to influence serum HBV DNA levels in HBV-infected patients. Previous studies have indicated that the removal of N-linked glycosylation from HBV envelope proteins impairs HBV virion secretion, contributing to immune escape and diagnostic failure [3, 6, 18, 45]. Here, we investigated the role of amino acid substitution at N-linked glycosylation site 320 by proline (N320P) in HBV replication and virion secretion. Seventy-two hours after transfection of HepG2.2.15 cells, the supernatants were collected, and the cells were lysed. Subsequently, replicative intermediates of HBV DNA from core particles observed in the cytoplasm and HBV DNA from virus particles secreted into the culture medium were analyzed by real-time PCR. The results demonstrated that the LHBs-N320P mutant did not exhibit significantly different intracellular HBV DNA levels compared to the wild type (Fig 6A). However, the LHBs-N320P mutant significantly increased HBV virion secretion compared with both the wild-type LHBs and LHBs-N320Q mutant (Fig 6B). The N320Q mutation (equivalent to N146 of SHBs) has been reported as an immune escape mechanism by impairing HBV virion secretion and was included in this study. The findings revealed that the LHBs-N320Q mutant could enhance intracellular HBV DNA levels while considerably impairing virion release into the growth medium. Taken together, these results indicate that replacing the amino acid at position 320 of the N-glycosylation site with proline enhances the release of HBV virions, thereby facilitating their immunological detection.

## Discussion

Hepatitis B is recognized as one of the most prevalent and severe infectious diseases globally, contributing substantially to morbidity and mortality rates. Approximately one-third of the global population is affected by HBV infection [46]. The detection of HBsAg holds paramount importance in the clinical diagnosis of HBV infection. Consequently, the emergence of antigenic escape mutants of HBsAg has significantly impacted vaccine development and diagnostic strategies [23].

The HBV surface proteins, crucial for assembling infectious virions, carry a single N-glycosylation site within the antigenic loops of the "a" determinant. This glycosylation pattern remains conserved across genotypes and infections [3]. Mutations or variants involving amino acid substitutions at this conserved N-linked glycosylation site, N320 of LHBs, have been detected in patients with chronic HBV infection [6, 18, 22]. Amino acid mutations at the conserved N-linked glycosylation site can potentially impact HBV protein expression, distribution, DNA replication, and virion secretion [22, 45, 47].

In this study, we generated different single amino acid-substituted mutants of LHBs at the conserved N-linked glycosylation site (N320) within the "a" determinant, including N320P, N320C, N320K, and N320Q, to assess their effects on antigenicity. Western blot and immunofluorescence analyses were conducted. Interestingly, the expression of the LHBs mutant protein in HEK-293T cells differed from that of wild-type LHBs. Notably, the N320P LHBs-mutated protein exhibited a significant increase in binding capacity with the specific antibody, followed by the N320Q and N320C LHBs-mutated proteins, compared to the wild-type, whereas the N320K LHBs-mutated protein showed reduced reactivity with the specific antibody compared to the wild-type. These findings suggest that amino acid mutations in the "a" determinant region, particularly at the conserved N-linked glycosylation site (N320), may impact the antigenicity of LHBs. However, these results reflect the outcomes of assays using specific antibodies that target the preS1 domain. For greater accuracy, antibodies specifically designed to target the mutation site will be essential.

Substitution of key amino acid residues within the critical region may influence their stability and structure, as well as their characteristics and interactions with other proteins [48]. Conformational analysis of LHBs variants might offer valuable insights into the structural basis of LHBs variation at the molecular level and elucidate how it impacts LHBs protein recognition by its specific antibody [49]. In this study, we utilized the I-TASSER server to model the 3D structures of both wild-type LHBs and mutants, followed by refinement using the GalaxyRefine server. The selection of the best refined model was based on the results obtained from the Ramachandran plot analysis. The findings revealed that mutations of the "a" determinant loop at N320P, N320C, N320K, and N320Q could impact the proper folding of LHBs and induce local conformational changes in the "a" determinant region. However, the N320K mutation exerted a more substantial effect on the conformation of the "a" determinant. Besides inducing local changes in the "a" determinant, the N320K mutation could also transform the alpha-helix into a non-repetitive secondary structure in the "a" determinant region compared to wild-type LHBs. Moreover, considering the properties of each amino acid, it is important to note that proteins are macromolecules composed of monomeric amino acids. Each amino acid possesses distinct properties attributable to its side chain, and the overall structure of a protein is influenced by its amino acid composition [50]. Asparagine (N), glutamine (Q), and cysteine (C) are uncharged amino acids with a polar side chain, whereas proline (P) is an amino acid with a non-polar side chain. On the other hand, lysine (K) is an amino acid with a positively charged side chain [51]. Thus, differences in the physicochemical properties of amino acids may affect the tertiary structure of the protein and alter the steric hindrance between other residues [52]. These findings indicate that changes in the reactivity of mutated LHBs protein, resulting from a single amino acid substitution at the conserved N-linked glycosylation site (N320), with a specific antibody may be attributed to conformational structural alterations.

Previous research has revealed that the antigenic determinations of the HBsAg are remarkably conformation-dependent. Thus, any modification in the “a” determinant region may influence the antigenic index of this protein [53]. In our study, we observed that a mutation in the "a" determinant region of LHBs at the asparagine position 320 led to alterations in protein antigenicity. Interestingly, our analysis using the IEDB server revealed that the N320P mutation resulted in an increase in LHBs antigenicity compared to both the wild-type and other variant sequences. Conversely, we found that the N320C mutation caused a significant reduction in LHBs antigenicity compared to the wild-type and other variants. However, the N320K and N320Q mutations resulted in only slight differences in antigenicity compared to the wild-type sequence. Notably, the changes in antigenicity observed for the N320P and N320K LHBs mutant proteins were strongly associated with the binding capacity of specific antibodies to these mutant proteins, as determined by western blotting and immunofluorescence staining.

Progress in immunoinformatics has been rapid, leading to the development of a variety of tools for predicting epitopes recognized by B-cells or T-cells, as well as antigenic responses [24]. Following the mutation at the conserved N-linked glycosylation site at position 320 to proline, the results showed an increase in the antigenic index at and around the mutation site compared to the wild-type sequence. Furthermore, we conducted additional investigations into the potential of the N320P mutation as a T-cell and B-cell epitope using *in silico* methods. The results of immunoinformatics analyses indicated that peptide sequences spanning residues 309–324 and 312–329 were predicted to be probable B-cell epitopes. Additionally, six peptides containing the N320P mutation were predicted to be probable T-cell epitopes, with positive immunogenicity scores. Moreover, the comparison of the final T-cell epitopes with linear B-cell epitopes revealed overlaps, indicating that some T-cell epitopic peptides were also present within B-cell epitopic peptides. This suggests that the N-linked glycosylation mutation at N320P plays a pivotal role as an immunogenic epitope. Nevertheless, the results of B-cell and T-cell epitope predictions using immunoinformatics necessitate further validation through both in vitro and in vivo studies.

Due to the overlapping arrangement of HBV ORFs, mutations occurring in the pre-S/S region can induce amino acid alterations in the overlapping P gene, particularly in HBV reverse transcriptase (RT). These mutations can lead to structural and functional modifications in RT, thereby potentially affecting viral replication and virion secretion [54–56]. Several studies have indicated that the elimination of a single-conserved N-linked glycosylation site within the "a" determinant region of the HBV envelope protein does not significantly impact the expression and secretion of HBs subviral particles. However, such mutations have been shown to disrupt virion secretion, potentially contributing to immune evasion. In this study, we explored the impact of substituting a single amino acid at the conserved N-linked glycosylation position, N320, of LHBs (equivalent to N146 of SHBs), with proline on HBV replication and secretion. Our observations revealed that the LHBs-N320P mutant did not significantly increase HBV DNA levels in HepG2.2.15 cells compared to the wild-type. Remarkably, the N320P mutant substantially promoted the secretion of progeny HBV DNA (in virions). Conversely, the N320Q mutant significantly enhanced HBV replication; however, the virions appeared to be trapped within the cells, resulting in impaired secretion into the cell culture supernatant compared to the wild- type. Indeed, virion secretion is contingent upon N-linked glycosylation at N146 within the S domain, but this process can be hindered by immune escape mutations [6]. Studies have shown that mutations at N146Q or N146S have the capacity to obstruct virion secretion, while subviral particle secretion remains unaltered [6, 18, 57].

In summary, our findings demonstrate that a single amino acid substitution at the conserved N-linked glycosylation site position N320, particularly by proline, can alter the antigenic reactivity of LHBs with specific antibodies, likely due to conformational changes. Moreover, the N320P mutation leads to an increased antigenic index at and around the mutation site, indicating its potential involvement in both B-cell and T-cell epitopes. Additionally, the mutation at the conserved N-linked glycosylation site at position N320 to proline significantly enhances the secretion of progeny HBV DNA in virions, potentially impacting HBV DNA diagnosis. However, further in-depth studies are needed to validate these findings. Overall, this study advances our understanding of viral immune evasion mechanisms, potentially leading to the development of enhanced diagnostic assays and vaccines against HBV.

## Supporting information

S1 Table Sequences of primers used in this study. (DOCX)

S1 Raw images Original uncropped immunoblot images. (PDF)

S1 Data Data relevant to the study. (XLSX)

S1 Fig Standard curve established for HBV DNA quantitation. (TIF)
