# Supplementary material for: In vitro and in silico analyses of amino acid substitution effects at the conserved N-linked glycosylation site in hepatitis B virus surface protein on antigenicity, immunogenicity, HBV replication and secretion
Source: PLoS One. 2025 Jan 6;20(1):e0316328. doi: 10.1371/journal.pone.0316328 (PMC11703054; doi:10.1371/journal.pone.0316328)
Supplement: S1 Table — (DOCX) [file pone.0316328.s001.docx]

| Primer sequences used in site-directed mutagenesis | |
| --- | --- |
| Name | **Primer Sequence (5’ to 3’)** |
| N320P | F: CATGTTGCTGTACAAAACCTACGGATGGACCCTGCACCTGTATTCCC |
|  | R: GGGAATACAGGTGCAGGGTCCATCCGTAGGTTTTGTACAGCAACATG |
| N320C | F: CATGTTGCTGTACAAAACCTACGGATGGATGCTGCACCTGTATTCCC |
|  | R: GGGAATACAGGTGCAGCATCCATCCGTAGGTTTTGTACAGCAACATG |
| N320K | F: GCTGTACAAAACCTACGGATGGAAAGTGCACCTGTATTC |
|  | R: GAATACAGGTGCACTTTCCATCCGTAGGTTTTGTACAGC |
| N320Q | F: TGTTGCTGTACAAAACCTACGGATGGACAGTGCACCTGTATTCC |
|  | R: GGAATACAGGTGCACTGTCCATCCGTAGGTTTTGTACAGCAACA |

| Primer sequences for detecting HBV DNA by real-time PCR | |
| --- | --- |
| Name | **Primer Sequence (5’ to 3’)** |
| HBV DNA | F: GTTGCCCGTTTGTCCTCTAATTC |
|  | R: GGAGGGATACATAGAGGTTCCTTGA |
