# Supplementary material for: In vitro and in silico analyses of amino acid substitution effects at the conserved N-linked glycosylation site in hepatitis B virus surface protein on antigenicity, immunogenicity, HBV replication and secretion
Source: PLoS One. 2025 Jan 6;20(1):e0316328. doi: 10.1371/journal.pone.0316328 (PMC11703054; doi:10.1371/journal.pone.0316328)

S1_Raw_images

Uncropped immunoblot images

Fig 1.

Repeat 1

Lane 1: protein ladder, Lane 2: wild-type, Lane 3: N320Q, Lane 4: N320P, Lane 5: N320C, Lane 6: N320K, Lane 7: pcDNA3.1, Lane 8: Non-transfected cells

LHBs protein (gp42 kDa and p39 kDa)

Lane 1 2 3 4 5 6 7 8

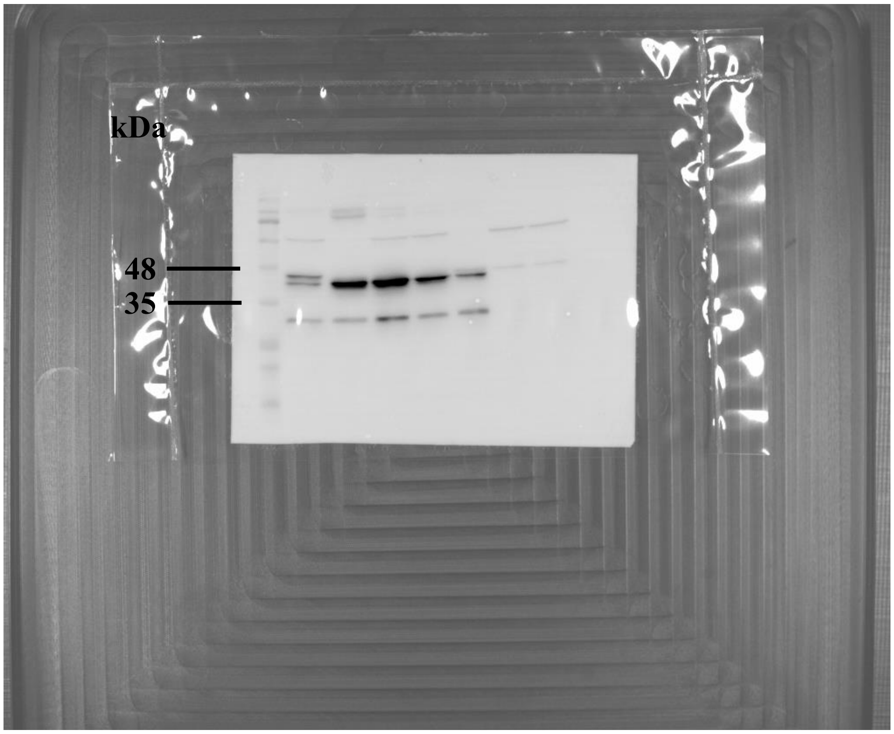

GAPDH loading control (37 kDa)

Lane 1 2 3 4 5 6 7 8

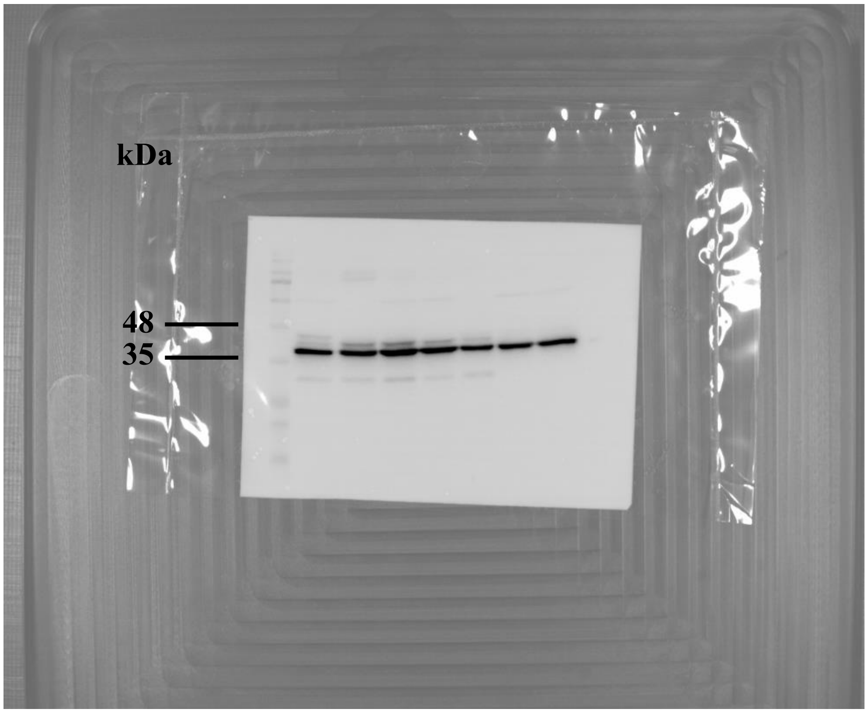

Fig 1.

Repeat 2

Lane 1: protein ladder, Lane 2: wild-type, Lane 3: N320P, Lane 4: N320C, Lane 5: N320K, Lane 6: N320Q, Lane 7: pcDNA3.1, Lane 8: Non-transfected cells

LHBs protein (gp42 kDa and p39 kDa)

Lane 1 2 3 4 5 6 7 8

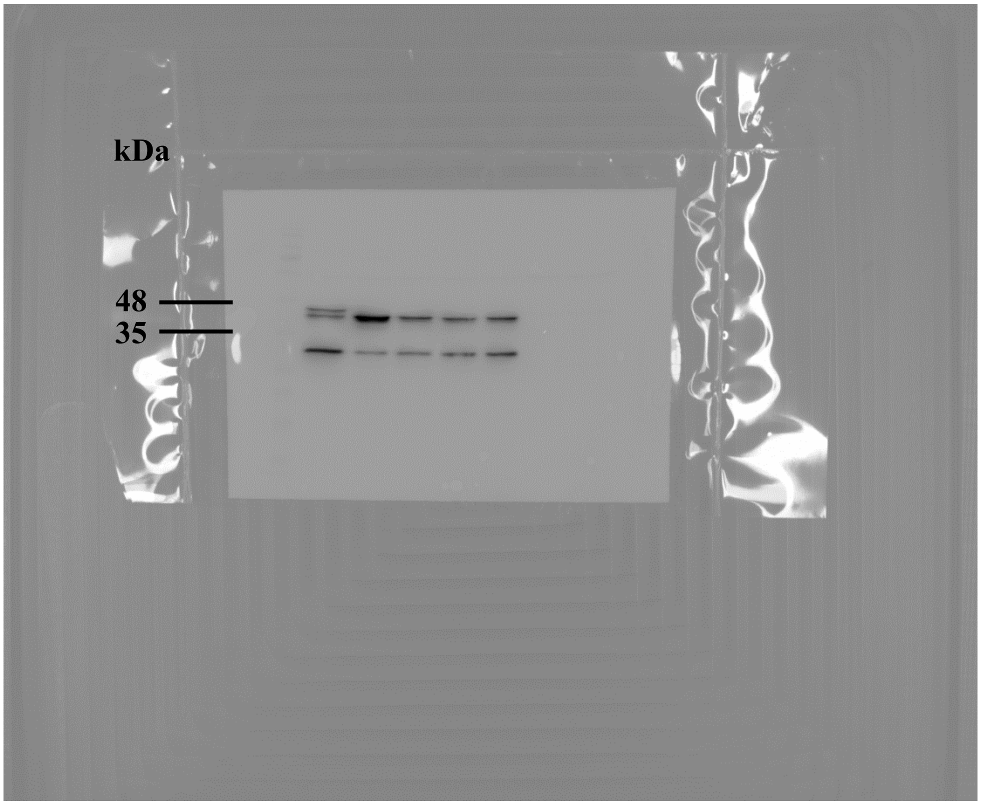

GAPDH loading control (37 kDa)

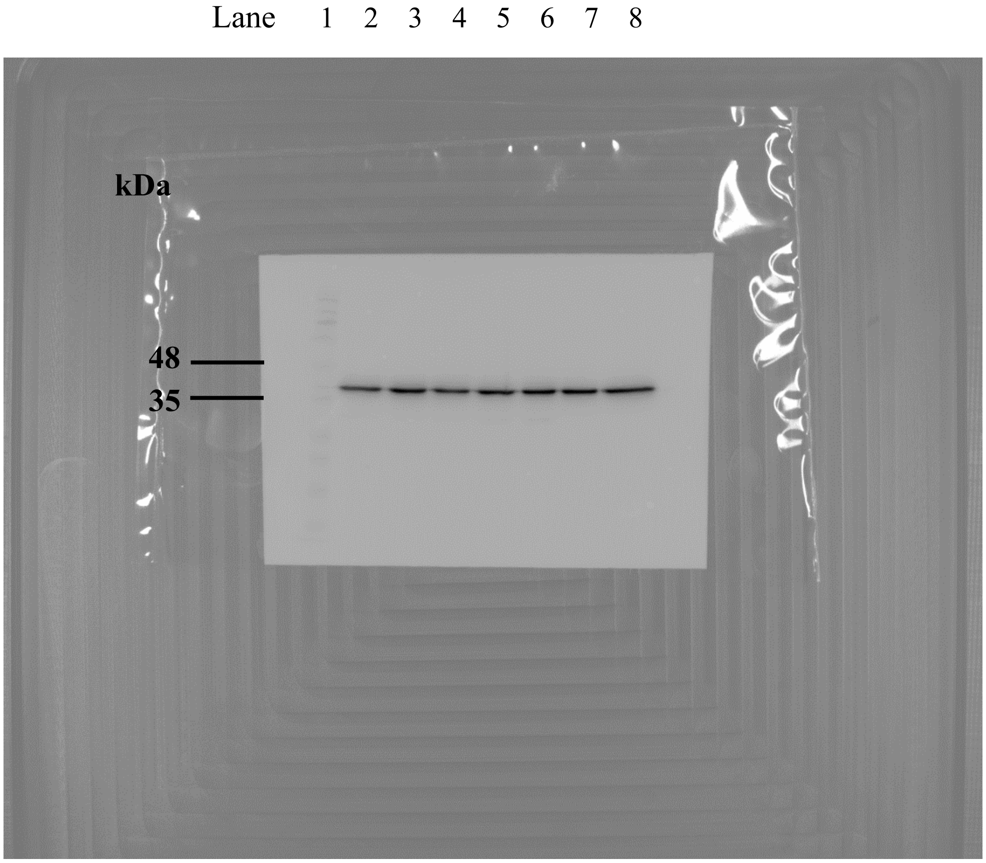

Fig 1.

Repeat 3

Lane 1: protein ladder, Lane 2: wild-type, Lane 3: N320Q, Lane 4: N320P, Lane 5: N320C, Lane 6: N320K, Lane 7: pcDNA3.1, Lane 8: Non-transfected cells

LHBs protein (gp42 kDa and p39 kDa)

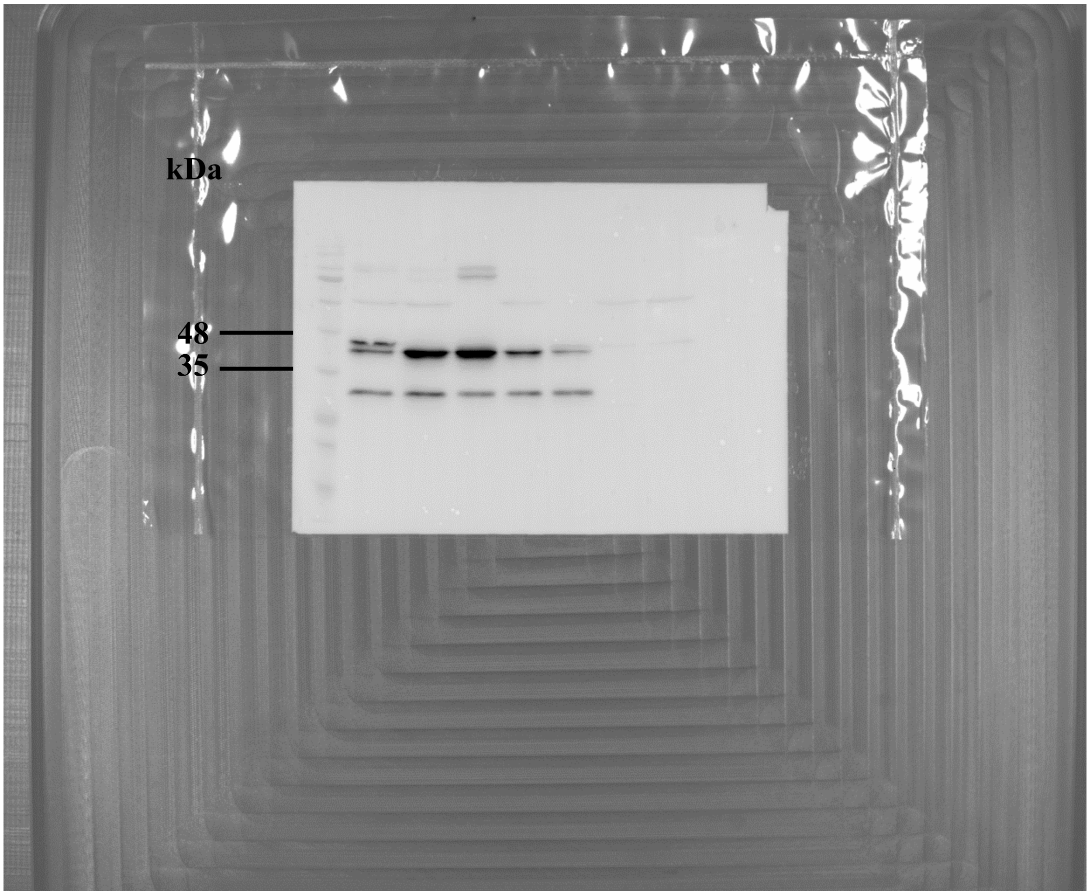

GAPDH loading control (37 kDa)

Lane 1 2 3 4 5 6 7 8

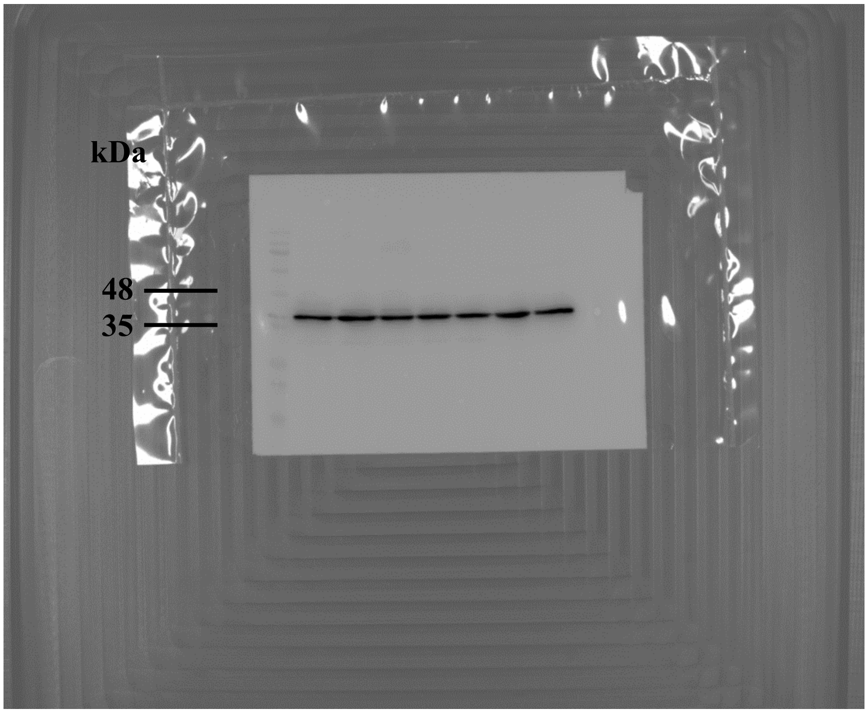

Supplement: S1 Raw images — (PDF) [file pone.0316328.s002.pdf]
