# Supplementary figures and images for: In vitro and in silico analyses of amino acid substitution effects at the conserved N-linked glycosylation site in hepatitis B virus surface protein on antigenicity, immunogenicity, HBV replication and secretion
Source: PLoS One. 2025 Jan 6;20(1):e0316328. doi: 10.1371/journal.pone.0316328 (PMC11703054; doi:10.1371/journal.pone.0316328)

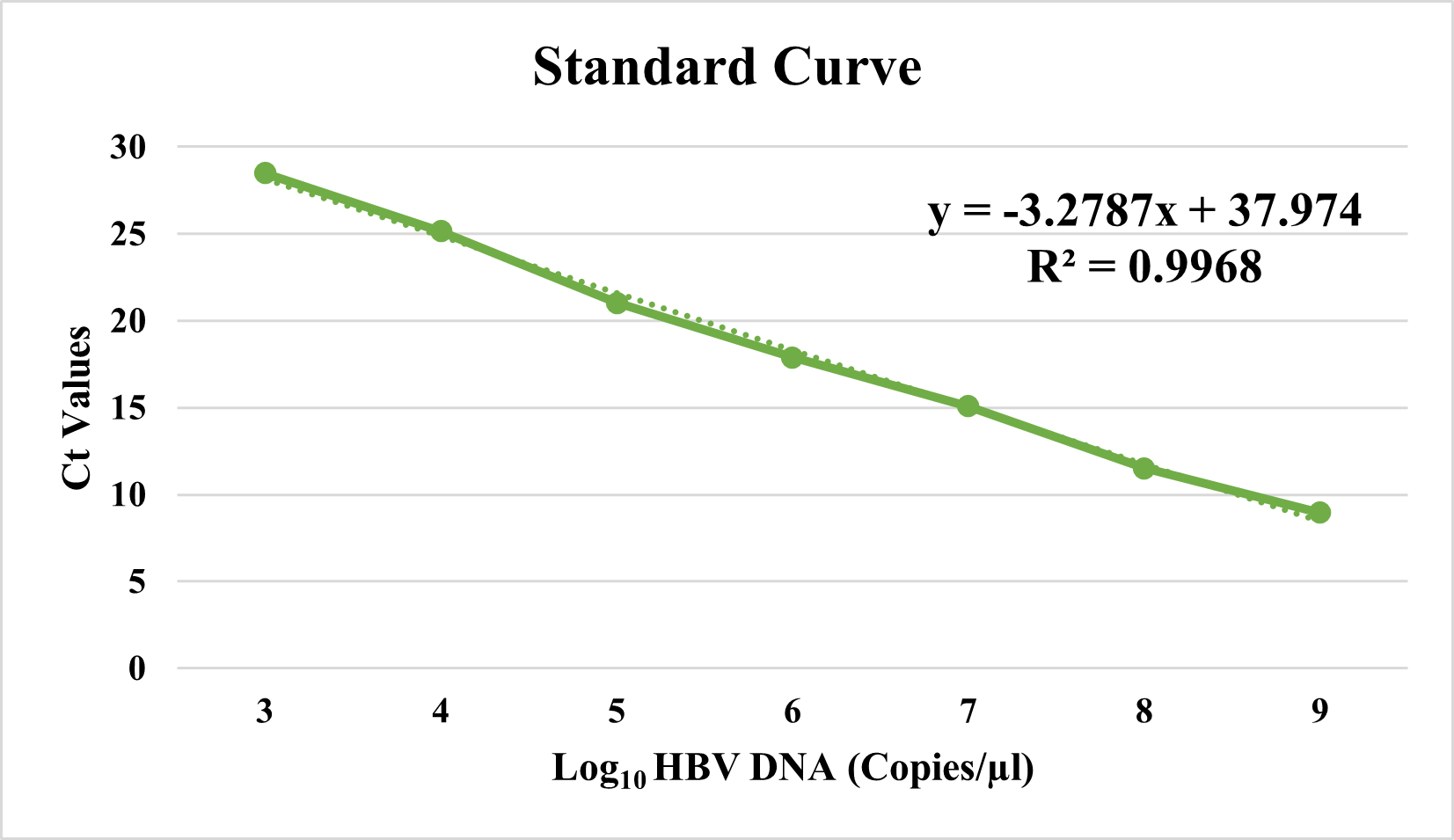

Supplement: S1 Fig — (TIF) [file pone.0316328.s004.tif]
